# Nuclear Transport of Yeast Proteasomes

**DOI:** 10.3389/fmolb.2019.00034

**Published:** 2019-05-16

**Authors:** Petra Wendler, Cordula Enenkel

**Affiliations:** ^1^Institut für Biochemie und Biologie, Universität Potsdam, Potsdam, Germany; ^2^Department of Biochemistry, University of Toronto, Toronto, ON, Canada

**Keywords:** proteasome, nuclear transport, importin, karyopherin, Blm10, proteasome storage granules

## Abstract

Proteasomes are key proteases in regulating protein homeostasis. Their holo-enzymes are composed of 40 different subunits which are arranged in a proteolytic core (CP) flanked by one to two regulatory particles (RP). Proteasomal proteolysis is essential for the degradation of proteins which control time-sensitive processes like cell cycle progression and stress response. In dividing yeast and human cells, proteasomes are primarily nuclear suggesting that proteasomal proteolysis is mainly required in the nucleus during cell proliferation. In yeast, which have a closed mitosis, proteasomes are imported into the nucleus as immature precursors via the classical import pathway. During quiescence, the reversible absence of proliferation induced by nutrient depletion or growth factor deprivation, proteasomes move from the nucleus into the cytoplasm. In the cytoplasm of quiescent yeast, proteasomes are dissociated into CP and RP and stored in membrane-less cytoplasmic foci, named proteasome storage granules (PSGs). With the resumption of growth, PSGs clear and mature proteasomes are transported into the nucleus by Blm10, a conserved 240 kDa protein and proteasome-intrinsic import receptor. How proteasomes are exported from the nucleus into the cytoplasm is unknown.

## Introduction

The yeast *Saccharomyces cerevisiae* is an amenable eukaryotic organism to study basic concepts of highly conserved cellular processes. One of these processes is the regulation of protein homeostasis by ubiquitin-dependent protein degradation in which the proteasome is engaged as key protease (Hershko and Ciechanover, 1998; Wolf and Menssen, 2018).

The proteasome is a multisubunit complex composed of ~40 different subunits. In yeast, proteasomal subunits are with few exceptions encoded by essential genes (Finley et al., 1998). Fourteen subunits belong to the proteolytic core particle (CP), also named 20S proteasome, which harbors the catalytic chamber within the interior of a barrel-shaped particle composed of four staggered rings. The outer rings are composed of seven distinct alpha (α) and the inner rings of seven distinct beta (β) subunits, yielding the CP with α_1−7_β_1−7_β_1−7_α_1−7_ configuration. Contemporaneously with the atomic resolution of the X-ray structure of the yeast CP (Groll et al., 1997), the CP was found to be assembled from two precursor complexes, namely half-CPs (Chen and Hochstrasser, 1996; Ramos et al., 1998). Five out of the seven β subunits are synthesized with propeptides which are autocatalytically processed. Three of the processed β subunits protrude an active site threonine into the interior of the CP. CP-dedicated chaperones, called Ump1 and Pba (alias Pac, Poc) 1–4, assist in CP subunit assembly. None of these CP-dedicated chaperones are essential (Budenholzer et al., 2017). Thus, CP assembly can occur by mass interactions as the proteasome is with approximately 0.5% of the total protein content and with ~2 × 10^4^ proteasomes per cell the second most abundant protein complex (Marguerat et al., 2012).

The proteasome achieves ~90% of the protein breakdown in growing yeast and cultured mammalian cells. Well-known examples of proteasomal substrates are short-lived proteins regulating cell cycle progression and gene expression, such as cyclins, cyclin-dependent kinases and their inhibitors, and transcriptional factors. Misfolded proteins, especially occurring during stress, are also proteasomal substrates of which some were found to be imported into the nucleus for proteasomal degradation (Wolf and Hilt, 2004; Gardner et al., 2005; Park et al., 2013; Tanaka, 2013). These proteins are marked as proteasomal substrates by poly-ubiquitin chains, which are formed by isopeptide bonds to lysine residues of the target protein and contain a chain of at least four isopeptide-bound ubiquitin molecules. Interestingly, lysine 48-linkage between the ubiquitin molecules is used for nuclear substrates suggesting that nuclear proteasomes prefer the conventional poly-ubiquitin chain with lysine-48 linked ubiquitin molecules. Cytoplasmic substrates have mixed lysine linkages which seem to be conferred by ubiquitin ligases associated with the ER (Samant et al., 2018). Whether proteasomes in the nucleo- and cytoplasm are differently configured to achieve ubiquitin-linkage specific degradation of proteasomal substrates needs to be investigated. There is an intriguing coincidence that proteasomes are primarily nuclear in dividing yeast and mammalian cancer cells (Enenkel, 2014b) suggesting that substrates with conventional lysine 48-linked poly-ubiquitin chains arise in the nucleus for proteasomal degradation (Samant et al., 2018).

Ubiquitin-dependent proteasomal proteolysis is an ATP-dependent process. How proteasomal substrates are degraded in an ATP-dependent manner is visualized by single particle cryo-electron microscopy analyses (de la Peña et al., 2018; Dong et al., 2018). These recent studies reconcile countless previous studies going back to the original discovery of ubiquitin in ATP-dependent proteolysis by Hershko and Ciechanover in the 1980ies, a discovery awarded with the Nobel Prize in 2004 (Hershko et al., 2000).

To recognize poly-ubiquitylated proteins, the proteasome requires the regulatory complex (RP) composed of ~ 23 different subunits which are assigned to two subcomplexes, the base and the lid. One or two RPs flank the CP by forming 26 and 30S proteasomes with single-capped RP-CP and double-capped RP-CP-RP configurations, respectively. The RP contains intrinsic ubiquitin receptors, Rpn10 and Rpn13, and docking sites for extrinsic ubiquitin receptors located at the base subunits Rpn1 and Rpn2. With a molecular mass of around 100 kDa Rpn1 and Rpn2 are the largest proteasomal subunits (Shi et al., 2016). The base complex also contains a ring of six ATPase subunits (Rpt1-6) which control the entry of proteasomal substrates into the CP catalytic chamber and confer ATP-dependence on protein degradation. In free CP, the N-termini of the α subunits close the entry in the middle of the α ring. By attachment of the RP ATPase ring, the gates are opened and committed to accept unfolded protein substrates (Groll et al., 2000). The poly-ubiquitin chain is removed by an isopeptidase activity conferred by the lid subunit Rpn11 adjacent to the ATPase ring, which triggers the unfolding and translocation of the polypeptide into the proteolytic cavity of the CP.

In yeast, two conserved high molecular mass proteins, named Blm10 (the ortholog of mammalian PA200) and Ecm29, are involved in the quality control of proteasome assembly. Blm10 preferentially binds mature CP with constitutively open α rings (Fehlker et al., 2003; Lehmann et al., 2008). One α ring bound to Blm10 results in hyperactive Blm10-CP as measured by the cleavage of chromogenic peptides (Schmidt et al., 2005). Both α rings bound to Blm10 results in Blm10-CP-Blm10 with repressed activity (Fehlker et al., 2003; Lehmann et al., 2008). Ecm29 binds proteasome holo-enzymes with defective subunit composition and impaired proteolytic activity (Kleijnen et al., 2007; Lehmann et al., 2010).

Proteasome configurations can be analyzed by native gel electrophoresis in lysates of cells expressing GFP-labeled proteasomes. GFP imaging of the native gels allows assigning proteasome configurations to distinct bands, since RP-CP-RP, RP-CP, their hybrids with Blm10 and Ecm29, free CP and RP migrate with different electrophoretic mobilities (Enenkel, 2012). During cell proliferation the ATP levels are high and proteasomes primarily exist as holo-enzymes. The ATP levels must be kept high during cell lysis and electrophoresis to stabilize proteasome holo-enzymes. Free CP might not be detectable, since the α-rings are closed yielding latent enzyme activity (Groll et al., 1997). The addition of 0.02% sodium dodecyl sulfate opens the α rings that chromogenic peptides can diffuse into the CP cavity (Orlowski and Wilk, 2000).

So far, we cited original and most recent work about the structure and function of yeast proteasome, though invaluable work was done over the last three decades and cannot be considered in this review due to limited space. With regard to our topic of this review, the nuclear import of proteasomes in yeast, we will provide a comprehensive overview of the literature.

## Nucleocytoplasmic Transport through the Nuclear Pore Complex

**Figure 1:**
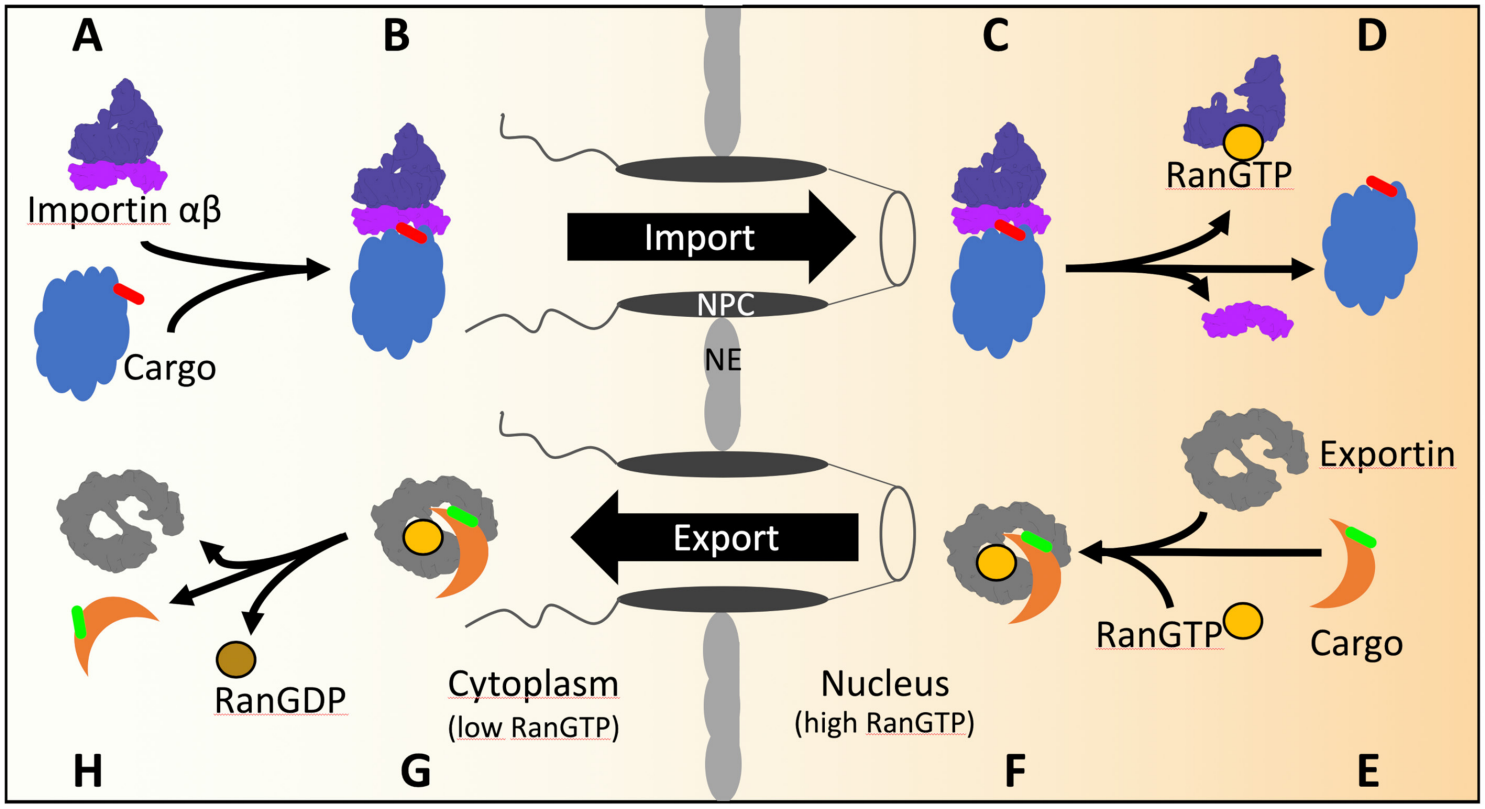
The transport cycles of importins and exportins in yeast. **(A)** Import of cNLS (red) containing cargos is mediated by importin αβ. **(B)** Importin α recognizes and binds the cNLS of the cargo as well as importin β. After transport through the NPC **(C)** the GTP bound GTPase Ran binds to importin β and dissociates the import complex **(D)**. **(E)** The export of NES (green) containing cargo from the nucleus is mediated by exportin. In the presence of GTP bound Ran, exportin binds Ran and the cargo **(F)** and transports the complex across the NPC into the cytoplasm **(G)**. Hydrolysis of GTP to GDP is stimulated by Ran binding protein 1 and Ran GTPase activating protein 1 in the cytoplasm. Conformational rearrangements in RanGDP lead to the dissociation of the exportin/RanGDP/cargo complex **(H)**.

In yeast, macromolecules are exchanged between the nucleo- and cytoplasm through the nuclear pore complex (NPC) studded throughout the nuclear envelope (NE) (Figure 1) (Wente and Rout, 2010; Aitchison and Rout, 2012). Intrinsically disordered phenylalanine-glycine (FG)-rich nucleoporins within the NPC build a selective sieve which is wide enough for passive diffusion of proteins of up to ~40 kDa or a diameter of 5 nm (Ribbeck and Görlich, 2002). Protein translocation through the NPC either by passive or receptor-mediated transport also depends on the surface amino acid composition. Acidic amino acids impede, while arginine, histidine, cysteine and hydrophobic residues facilitate NPC passage (Frey et al., 2018). How these structural requirements are met in protein complexes like the proteasome and how they confer directionality to nuclear transport is unknown. Effective translocation of protein complexes requires active transport using transport receptors that facilitate the directed trafficking of cargo proteins through the NPC. These transport receptors recognize localization signals within the cargo protein which determine the final destination. The NLS (nuclear localization sequence) and the NES (nuclear export sequence) mark the protein cargo for nuclear import and export, respectively. In yeast, 13 NLS-specific and one NES-specific transport receptors are known (Wozniak et al., 1998; Macara, 1999). Nuclear transport receptors, alternatively named karyopherins or importins/exportins, are members of the β-karyopherin family, a subclass of the HEAT-(**H**untingtin, elongation factor 3 (**E**F3), protein phosphatase 2A (PP2**A**), PI3-kinase **T**OR1) repeat family.

The gradient of the small GTPase Ran across the NE confers directionality of nuclear transport through the NPC. Ran GTPase, in yeast named Gsp1, is GTP-bound in the nucleoplasm and GDP-bound in the cytoplasm, as a result of the activities of nuclear Ran guanine nucleotide exchange factor and cytoplasmic RanGTPase activating protein (Görlich and Kutay, 1999). During import, the importin assembles with the cargo in the cytoplasm. Arrived in the nucleus the importin-cargo complex encounters RanGTP (Figures 1A–D) which triggers the release of the cargo into the nucleus and the return of importin-RanGTP to the cytoplasm. In the cytoplasm, the RanGTPase activating protein stimulates RanGTP hydrolysis and recycles importin for the next round of nuclear import. During export, the binding of RanGTP to the exportin stabilizes the interaction of the exportin with the cargo during the passage through the NPC into the cytoplasm (Figures 1E–H). Upon RanGTP hydrolysis in the cytoplasm the cargo is released from the exportin (Görlich and Kutay, 1999).

The importin/karyopherin αβ heterodimer, named Srp1/Kap95 in yeast (Enenkel et al., 1995), was the first discovered import receptor, which founded the canonical / classical pathway using the canonical/classical NLS (cNLS). The cNLS are classified as either monopartite or bipartite cNLS. The prototype of the monopartite cNLS is found in the SV40 large T-antigen (PKKKRKV) and consists of a short stretch of basic amino acids. The prototype of the bipartite cNLS consists of two clusters of basic amino acids that are separated by a spacer of about 10 amino acids and is present in the nucleosome assembly chaperone nucleoplasmin (KR[PAATKKAGQA]KKKK) (Dingwall and Laskey, 1991).

## How Proteasome Localizations Depend on the Growth Conditions in Yeast

Epitope tagging of chromosomal proteasomal genes with GFP allows for *in vivo* localization studies of proteasomal subunits at endogenous expression levels. Gradient ultracentrifugation confirmed that the fraction of free GFP-tagged subunits is negligible compared with the fraction of GFP-tagged subunits incorporated into proteasomes. Each GFP-tagged version of a CP or RP subunit shows the same intracellular, nuclear localization in dividing yeast cells (Laporte et al., 2008). With the transition from cell proliferation to quiescence, a reversible state of non-proliferation induced by glucose depletion, proteasomes migrate to the NE. Electron microscopy of immunogold-labeled proteasomes revealed proteasomes to be accumulated at the nucleoplasmic side of the NE (Wilkinson et al., 1998). The high molecular mass protein Esc1 (Establishes silent chromatin) was found to anchor the RP to the nuclear basket of the NPC (Niepel et al., 2013). This finding supports Günter Blobel's gene gating hypothesis (Blobel, 1985) and follow-up models that call for proteasome functions in chromatin silencing and remodeling, the special regulation of transcriptional activities, DNA repair and the maintenance of the NPC (Nagai et al., 2011). RP subunits were also found to be associated with Ran-binding protein 2 (Ran BP2), alternatively named Nup358, which is part of the cytoplasmic fibers of the NPC (Ferreira et al., 1998).

Biochemical fractionations revealed RP subunits in association with the NE and NPC. The presence of detergents necessary for NE and NPC preparations impacts the stability of proteasome holo-enzymes. Cryo-electron tomography, a non-invasive technique to visualize the *in situ* localization of macromolecules within the cell, revealed proteasome holo-enzymes attached to the NPC (Albert et al., 2017).

In prolonged quiescence the ATP level declines due to the shortage of nutrients (Laporte et al., 2011) and proteasome holo-enzymes dissociate into CP and RP (Bajorek et al., 2003). In yeast, cell cultures grown to stationary phase are depleted for glucose and contain quiescent cells. Proteasome storage granules (PSGs) start to form at the NE where they pinch off into the cytoplasm as originally described by Isabelle Sagot and her co-workers (Laporte et al., 2008). According to her electron microscopic studies PSGs are membraneless and presumably represent new members of the growing family of liquid organelles (Shin and Brangwynne, 2017). Our mass spectrometry analysis of cross-linked PSGs suggests that PSGs are densely packed with proteasomes. If quiescent wild type cells in which proteasomes are sequestered into PSGs are analyzed by native page electrophoresis, proteasomes are dissociated into Blm10-capped CP and RP (Gu et al., 2017).

Interestingly, these PSGs are highly mobile within the cytoplasm and clear with the exit from quiescence by the addition of glucose that signals the resumption of growth and the immediate relocation of proteasomes into the nucleus.

In principle, two major nuclear import pathways are known for yeast proteasomes. One pathway describes how precursor complexes are imported into the nucleus, specifically in highly proliferating cells, when precursor complexes are continuously assembled from newly synthesized subunits in the cytoplasm. In the other pathway holo-enzymes are imported upon the exit from quiescence, when precursor complexes are not readily available. Since the transition from proliferation to quiescence is fluent, both import pathways may coexist with different flux depending on the metabolic state. All components in nuclear import of proteasomes are conserved from yeast to human suggesting that similar import pathways exist for yeast and mammalian proteasomes despite the fact that yeast cells having a closed mitosis exchange proteins between the nucleo- and cytoplasm only through the NPC. Alternative import pathways have to be considered in those mammalian cells, in which the NE disassembles and reassembles during mitosis. Accordingly, constituents of the nucleo- and cytoplasm are exchanged during open mitosis (Groothuis and Reits, 2005).

## Nuclear Import of the CP in Proliferating Yeast

From a simplified view, the CP is assembled from two half-CP precursor complexes in which certain β-subunits have β-pro-peptides. Five CP-dedicated chaperones, named Ump1 and Pba 1-4, assist in subunit incorporation with Ump1 having a pivotal role in CP maturation. In the cytoplasm the CP assembly starts with the formation of α rings from newly synthesized α subunits which serve as template for β subunit incorporation. Ump1 and β7, one of the last subunits to be incorporated, guide the rate-limiting step of the dimerization of two half-CP precursor complexes into the pre-holo-CP, an unstable assembly intermediate. Within the pre-holo-CP the β-pro-peptides are autocatalytically processed by exposing the active site threonines of three β-subunits. Ump1 buried in the interior of the pre-holo-CP becomes the first substrate of the nascent CP (Ramos et al., 1998). Despite its small molecular mass Ump1 (17 kDa) can be functionally tagged with GFP (25 kDa) which allows monitoring its localization in living yeast cells and the isolation of Ump1-containing precursor complexes. Ump1-containing precursor complexes still lack the last incorporated β7 subunit (Lehmann et al., 2002) which in the matured CP embraces the opposing half-CP with its C-terminal region (Marques et al., 2007). Most importantly, Ump1 as well as its mammalian homolog hUmp1 is localized in the nucleus (Lehmann et al., 2002; Hoefer et al., 2006).

During CP maturation major conformational changes not only occur in Ump1 (Kock et al., 2015), a protein with intrinsically disordered domains (Sá-Moura et al., 2013; Uekusa et al., 2014). Within Ump1-containing precursor complexes, the α- and β-rings are loosely packed with wider α-ring openings than in the mature CP. The wider α ring opening allows the partial embedding of the Pba1-Pba2 heterodimer. Upon the maturation of the catalytic chamber the Pba1-Pba2 heterodimer is expelled from the α ring. The α rings undergo conformational changes from an open toward a closed central pore which controls the access of protein substrates into the mature CP (Kock et al., 2015).

Until 2000 reconstitution experiments using digitonin-treated mammalian cancer cells in which plasma membranes are partially permeabilized while NE remain intact, were used to study nuclear import of proteasomes, specifically of the *Thermoplasma acidophilum* CP. The *Thermoplasma acidophilum* CP has a simple α_7_β_7_β_7_α_7_ configuration with identical α and β subunits. The subunits can be expressed as recombinant proteins, assembled and processed into mature CP without the aid of CP-dedicated chaperones. Then, the *in vitro* assembled CP was chemically labeled with fluorescein dyes and studied in reconstitution assays of nuclear import using digitonin-permeabilised mammalian cells. Despite the origin from an akaryotic organism, the *Thermoplasma acidophilum* CP was slowly imported into the nucleus (Wang et al., 1997; Enenkel, 2014a). Since the central pore of the NPC can accommodate protein cargoes with a diameter of up to 39 nm (Panté and Kann, 2002), even proteasome holo-enzymes with RP-CP-RP configuration and an estimated diameter of 15 nm and length of 45 nm (Förster et al., 2013) can pass the NPC in longitudinal direction. However, effective nuclear import of macromolecules with these dimensions depends on Ran GTPase and specific NLS, which are recognized by cognate transport receptors. Proteasome holo-enzymes loaded with extrinsic ubiquitin-receptors and poly-ubiquitylated substrates might be too bulky to translocate easily through the NPC.

**Figure 2:**
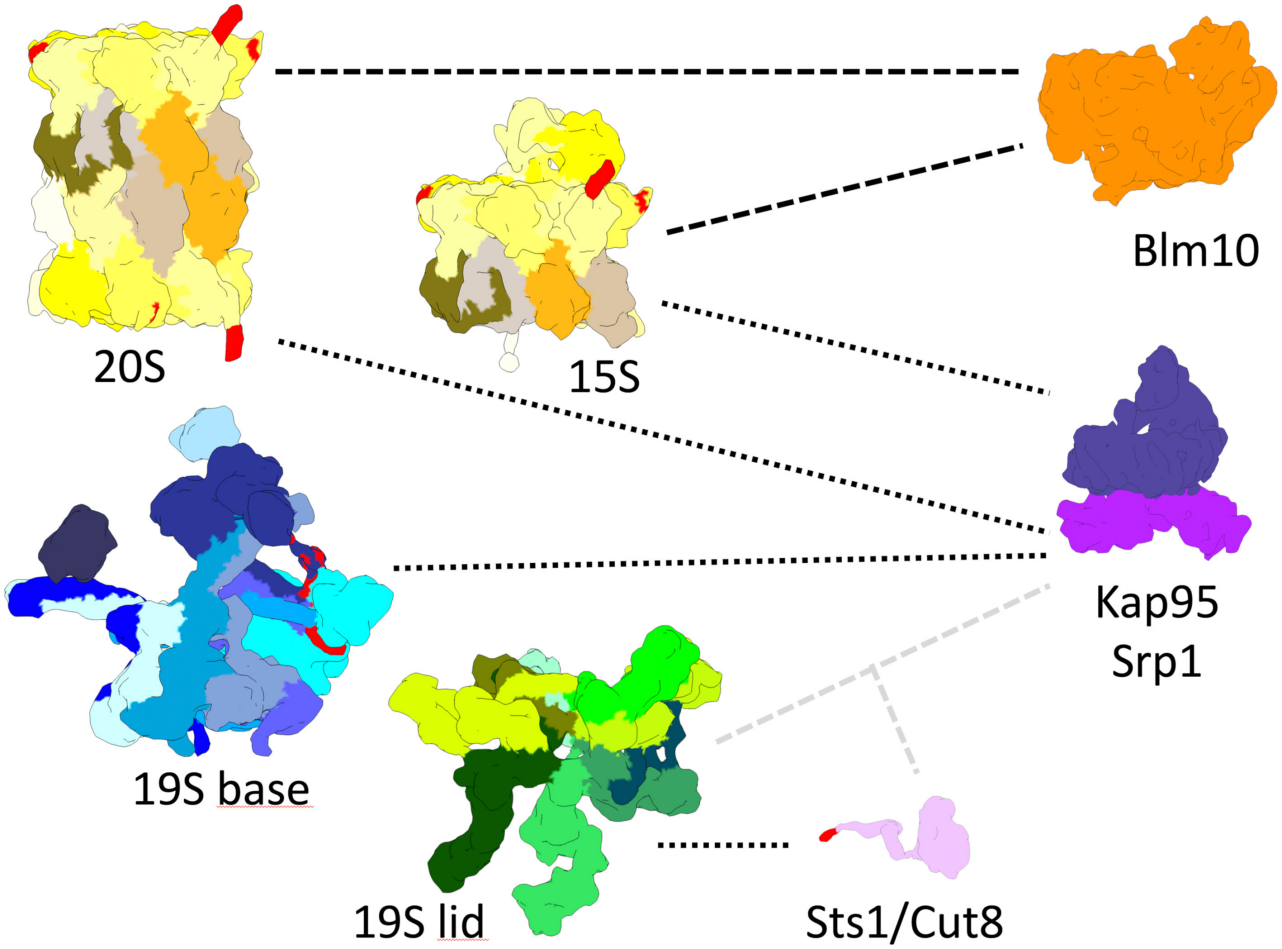
True-to-scale representation of proteasomal subcomplexes, NLS conferring adaptor proteins and nuclear transport receptors. The 20S CP, Pba1-2 bound 15S precursor complex, 19S base and 19S lid complexes are depicted with putative cNLS in α1, α4, α5, Rpn2, and Rpt2 colored in red. Importin αβ (Srp1/Kap95) binds to 20S, 15S, and 19S base complexes during nuclear import. Sts1/Cut8 binds to the 19S lid complex and possibly provides a cNLS, depicted in red, for importin αβ dependent nuclear import. Blm10 can bind to 20S and 15S complexes and possibly acts as an import receptor for nuclear transport.

Putative cNLS of the SV40 large T-antigen prototype are present in α-subunits of yeast and human CP (Figure 2). These proteasomal cNLS when fused to non-nuclear proteins such as fluorescein-labeled albumin, promoted the import of the fusion proteins into the nucleus of digitonin-permeabilised mammalian cells. The interpretation of these findings was that the nuclear import of the CP follows the rules of the canonical pathway (Nederlof et al., 1995; Knuehl et al., 1996). Notably, the α subunit of the *Thermoplasma acidophilum* CP has a KKVRSR sequence which conforms with a cNLS. The CP mutant with truncated cNLS was not imported into the nucleus (Wang et al., 1997). Fluorescent labeled human CP having four putative cNLS was also imported into digitonin-permeabilized mammalian cells though independent of importin αβ (Mayr et al., 1999). Three of the proteasomal cNLS are conserved from yeast to humans. According to the X ray structure of the yeast CP putative cNLS are accessible in the C-terminal region of the α subunits 1, 4 and 5, respectively (Lehmann et al., 2002). In human CP cNLS are present in subunits α1-4 which were verified to be functional as cNLS fusion proteins (Nederlof et al., 1995; Knuehl et al., 1996; Wu et al., 2018).

As early as 1990 Keiji Tanaka already proposed that proteasomal cNLS are not always accessible (Tanaka et al., 1990) which may have lead to inclusive results. The CP was proposed to occur in two conformations with either accessible or masked cNLS depending on tyrosine phosphorylation (Tanaka et al., 1990). Accordingly, the CP exists either in an import-competent or import-incompetent state.

At that time *srp1* mutants in importin α became available (Yano et al., 1992) The *srp1-31* allele was described as deficient in nuclear import of cNLS containing proteins and displayed the phenotype of proteasomal mutants with impaired degradation of cyclin Clb2 and cell cycle arrest (Loeb et al., 1995; Shulga et al., 1996). Subsequent studies showed that *srp1-49* but not *srp1-31* mutants are defective in the degradation of proteasomal substrates (Tabb et al., 2000). We confirmed this phenotype for the *srp1-49* (S116F) but not the *srp1-31* (E145K) allele, after we had requested the original strains from Masayasu Nomura (Yano et al., 1992). We sequenced both alleles and verified the mutations, respectively.

**Figure 3:**
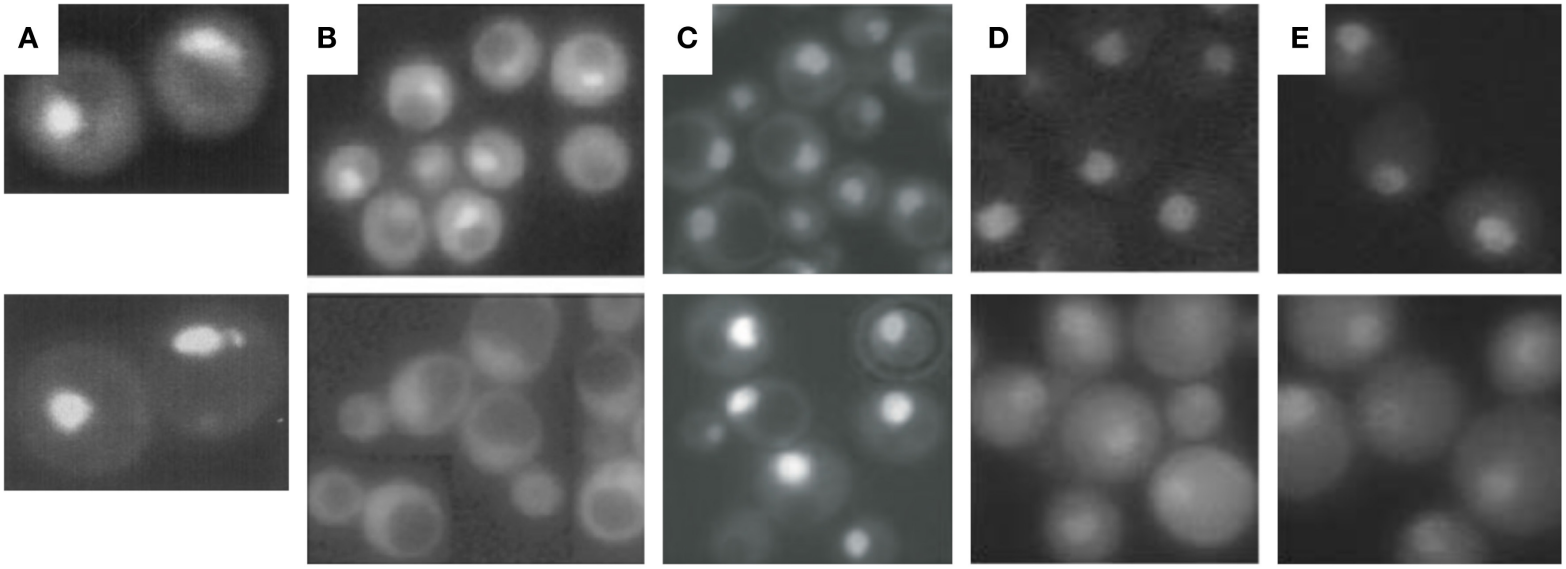
Cellular localization of proteasomal subunits. **(A)** Direct fluorescence microscopy of yeast wild-type cells expressing GFP-labeled Ump1 instead of the endogenous protein (top panel) and DAPI stained nuclei. **(B)** Fluorescence microscopy of GFP-labeled Ump1 in SRP1 wild type (top panel) and *srp1-49* cells grown at permissive temperature (lower panel). The research in **(A,B)** is reprinted from Lehmann et al. (2002). **(C)** Fluorescence microscopy of GFP-tagged β5 in wild-type (top panel) and *ump1*Δ cells (lower panel). This research was originally published in EMBO reports (Lehmann et al., 2008). **(D)** Fluorescence microscopy of GFP-tagged Rpn11 in SRP1 wild-type (top panel) and *srp1-49* cells grown at restrictive temperature (lower panel). **(E)** Fluorescence microscopy of GFP-tagged Rpn1 in SRP1 wild-type (top panel) and *srp1-49* cells grown at restrictive temperature. The research in **(D,E)** was originally published in Wendler et al. (2004).

Few years later in the middle of the 1990ies, the basic concept of CP biogenesis was understood and helped us in the interpretation of nuclear proteasome localization in yeast. Based on three major observation we proposed a model upon which the CP is imported into the nucleus as precursor complex rather than mature particle (Figure 3).

First, insight into nucleocytoplasmic transport of proteasomes has been facilitated by live cell imaging technologies using GFP reporter proteins such as of Ump1. Ump1-GFP was predominantly nuclear (Figure 3A) and trapped during β-pro-peptide processing, since unprocessed and incompletely processed CP subunit β5 were associated with Ump1. Importin α (Srp1)-containing import intermediates were also co-precipitated by tagged versions of proteasomal α subunits. They contained un- and incompletely processed β5 subunits suggesting that half-CP and pre-holo-CP are recognized by Srp1 (Lehmann et al., 2002).

Second, Ump1 and CP localizations were shifted toward the cytoplasm in Ran cycling and importin α mutants, specifically in the *srp1-49* (E145K) mutant (Figure 3B), suggesting that the canonical importin αβ receptor is responsible for nuclear import of proteasome precursor complexes (Lehmann et al., 2002).

We assumed that Srp1 recognizes proteasomal NLS differentially than cNLS prototypes (Chen and Madura, 2014b) supporting the early hypothesis that Srp1 has a dual role in cNLS-dependent import (Tabb et al., 2000). It still remains to be explained why nuclear targeting of proteasomes is deficient in *srp1-49* mutants which are import-proficient for proteins with cNLS prototypes (Loeb et al., 1995; Lehmann et al., 2002).

The third argument in favor of nuclear CP maturation is that most proteasomes remain nuclear when CP maturation is delayed by *UMP1* deletion (Figure 3C) (Fehlker et al., 2003). *UMP1* deletion results in ~50% completion of CP maturation which is compensated by induced proteasomal gene expression (Ramos et al., 1998). This regulatory feedback mechanism in *ump1*Δ cells is explained by impaired proteasomal degradation of the transcription factor Rpn4 resulting in augmented proteasomal transcripts (Dohmen et al., 2007). Thus, the expression of proteasomal genes is upregulated and the amount of CP precursor complexes doubled (Xie and Varshavsky, 2001; Dohmen et al., 2007). Considering the possibility that the CP is matured in the cytoplasm, incompletely matured CP should have been retained in the cytoplasm of *ump1*Δ cells. Instead, CP precursor complexes accumulate in the nucleus. Additionally, GFP-labeled Blm10 and Ecm29 which control late steps in proteasome assembly are hardly detected in wild type cells. Again, Blm10 and Ecm29 accumulate in the nucleus of *ump1*Δ cells supporting our conclusion that quality control of proteasome assembly is required in the nucleus (Lehmann et al., 2008, 2010).

However, since almost all experiments are carried out with some form of mutant strain or protein, we cannot rule out that transport of mature 20S proteasomes is possible in proliferating wt yeast cells. Proteasome maturation requires all subunits to come together in a spatially and temporally coordinated manner. Incorporation of the last subunit, β7, into the 15S complex triggers dimerization and propeptide cleavage in β1,2,5,6, and 7. While the 15S complex forms a stable intermediate, incorporation of β7 into the 15S is rate limiting for the very fast, last steps of proteasome maturation (Li et al., 2007; Marques et al., 2007). Deletions and even tags on proteasomal subunits might slow down proteasome maturation at arbitrary points. At which time point in proteasome maturation the complexes are transported across the nuclear envelope in proliferating wt yeast cells is most likely dependent on the rate of nuclear transport and the rate of proteasome assembly.

In contrast to yeast, mammalian proteasomes mature at the outer surface of the NE, the endoplasmic reticulum (ER), suggesting that proteasomes are taken up into the nucleus as matured enzymes (Fricke et al., 2007; Wu et al., 2018). The crystal structure of the CP-dedicated chaperone Pba1-Pba2 bound to the yeast CP showed that none of the NLS is masked by Pba1-Pba2 (Stadtmueller et al., 2012). Also early CP-dedicated chaperones Pba3-Pba4 that guide α ring assembly and may mask NLS (Takagi et al., 2014) are absent in Ump1-containing precursor complexes in yeast (Lehmann et al., 2002). Pba3-Pba4 localizes with early assembly intermediates in the cytoplasm and in the absence of Ump1 in the nucleus (Le Tallec et al., 2007). In mammalian cells, Pba1-Pba2 retains the α rings in to cytoplasm and prevents nuclear translocation of CP assembly intermediates regardless of the presence of NLS in α subunits (Wu et al., 2018). Thus, the mechanism by which Pba1-Pba2 prevents nuclear import of mammalian CP assembly intermediates is unclear.

Yeast spheroblasts soaked with fluorogenic peptide substrates showed proteasomal proteolysis *in situ* in the nuclear periphery (Enenkel et al., 1998). According to the model of Karim Madura proteolytically active proteasomes reside at the cytoplasmic surface of the NE. He postulates that proteasomes are stuck in the basket of the NPC and do not enter the nucleoplasm, since nuclear targeting in *srp1-49* mutants is impacted for proteasomes but not for cNLS-containing proteins (Chen and Madura, 2014a; Dang et al., 2016). Indeed, the nuclear protein Esc1, which is proposed to tether the proteasome to the NPC interactome, is anchored to the NE close to the nuclear basket and bound in almost stoichiometric amount to the RP and Ecm29 (Niepel et al., 2013). Whether NPC-anchored proteasomes are engaged in protein degradation remains to be tested, since Ecm29-associated proteasomes are inactive (Kleijnen et al., 2007; Lehmann et al., 2010).

*In situ* cryo-electron tomography in the native cellular environment visualized proteasome holo-enzymes seemingly engaged in the substrate-processing state to be crowded around the NE and NPC basket in *Chlamydomonas reinhardtii*, an unicellular organism with closed mitosis (Albert et al., 2017). An intact cytoskeleton also plays an important role in guiding proteasomes to the NPC (Cabrera et al., 2010), since the NPC is tethered to the cytoskeleton (Goldberg, 2017).

## Nuclear Import of the RP in Proliferating Yeast

To open the CP α ring for translocating an unfolded polypeptide substrate, the RP ATPases penetrate with their C-terminal HbYX motifs specific pockets between α subunits which anchor the RP ATPase ring to the CP α ring (Lander et al., 2012). Premature association of incomplete RP base assembly intermediates is prevented by four RP-dedicated chaperones, named Hsm3, Nas2, Nas6, and Rpn14, which obstruct the interface between the RP ATPase ring and the CP α ring, until the RP base is fully functional (Barrault et al., 2012). Aided by RP-dedicated chaperones recombinant RP subunits can be assembled into base and lid subcomplexes without the need of the CP as assembly platform (Besche et al., 2009). The maturation of the CP even induces an affinity switch that controls the association with the RP (Wani et al., 2015). Thus, RP and CP precursor complexes can be independently imported into the nucleus.

To yield CP and RP in equal stoichiometry in the nucleus, it is conceivable that the canonical importin αβ-dependent pathway is used for nuclear import of the CP and RP. Indeed, nuclear import of GFP-labeled Rpn1 and Rpn11, reliable reporters of RP base and lid, respectively, is significantly impaired in *srp1-49* mutants (Figures 3D,E; Wendler et al., 2004) as later confirmed by others (Chen and Madura, 2014b). All 13 available yeast mutants with either deletions or temperature-sensitive alleles of β-importins were tested for proteasome localisations. None of the null mutants in which non-essential importins were deleted showed cytoplasmic localisations of proteasomes in proliferating yeast (Wendler and Enenkel, unpublished work). Only *srp1-49* mutants were deficient in nuclear import of the RP suggesting that the essential importin αβ is responsible for an essential cargo such as the RP (Wendler et al., 2004). The cNLSs were found to be located in the N-terminal region of Rpt2 and the C-terminal region of Rpn2, which both belong to the RP base. GFP fusion proteins of the Rpt2 and Rpn2 NLSs were recognized by importin αβ and directed into the yeast nucleus. The functionality of both cNLS in the context of the incorporated subunits was further tested in yeast mutants. The RP base with truncations in either Rpt2 or Rpn2 NLS was not recognized by importin αβ. The Rpt2 cNLS was dispensable, while the Rpn2 cNLS was detrimental for nuclear targeting (Wendler et al., 2004). Later Erica Isono confirmed that Rpn2 contributes an essential cNLS to nuclear import of the RP base and that the RP lid is imported into the nucleus independently of the RP base. This finding supported our model that the RP is assembled from subcomplexes in the nucleus (Isono et al., 2007). Intriguingly, in *Xenopus* oocytes, Rpn2 and Kap95 were identified within an import-competent intermediate of proteasome assembly, providing yet another link between nuclear import and proteasome assembly (Savulescu et al., 2011). Both Rpn2 and Kap95 seem to fulfill synergistic functions in nuclear import (Huber and Groll, 2012).

**Figure 4:**
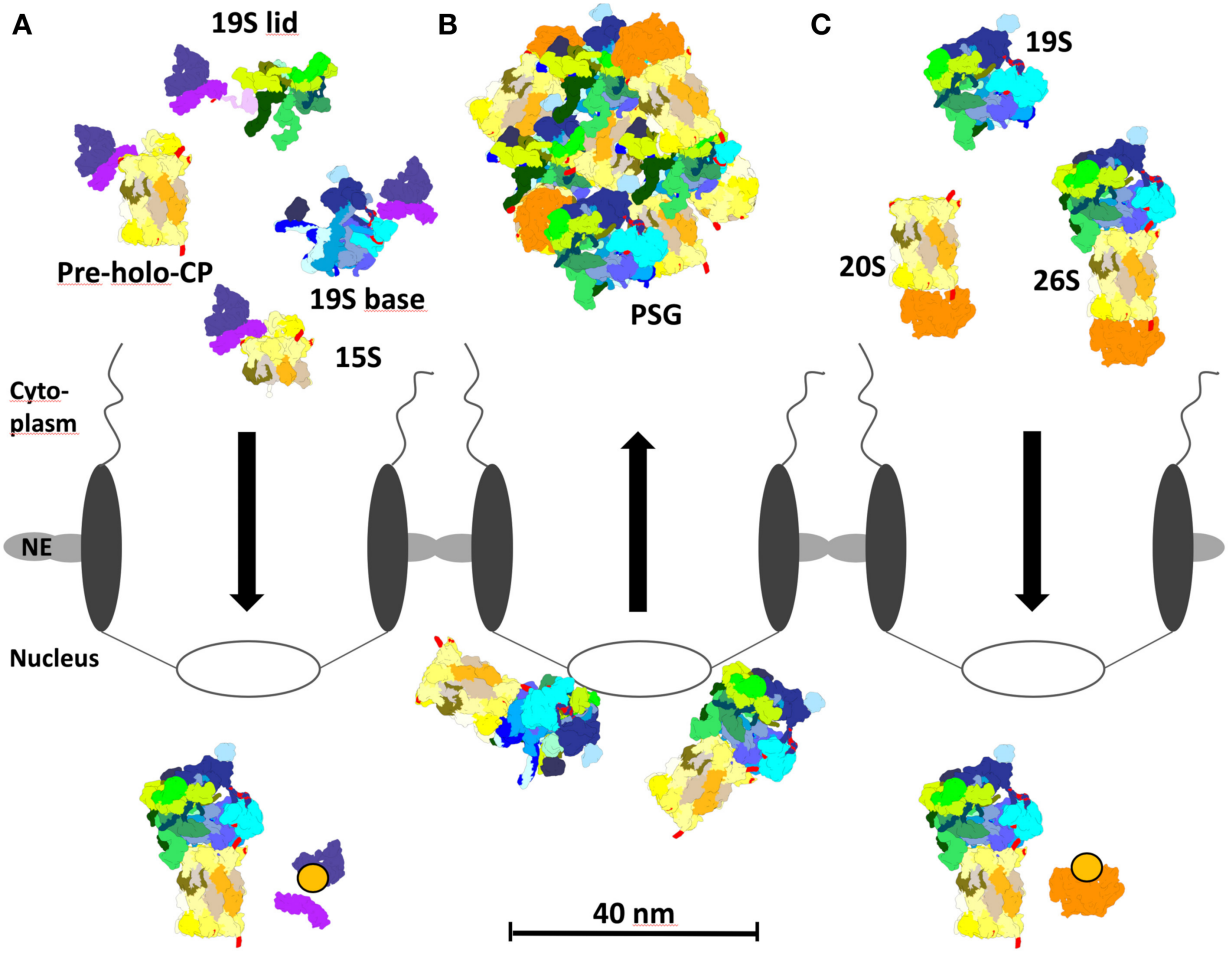
Proteasome localization during different growth phases in yeast. **(A)** In proliferating yeast, 15S precursor complexes, pre-holo-proteasomes, and 19S base complexes are imported into the nucleus via the cNLS pathway and importin αβ. Importin αβ binds to newly assembled proteasomal complexes in the cytoplasm. RanGTP dissociates the import complex in the nucleus, so that proteasome assembly and maturation can take place. **(B)** During quiescence, 26S proteasomes initially locate to the nucleoplasmic side of the NPC. Prolonged quiescence leads to the break up into 19S RP and 20S CP, which are sequestered by Blm10 to protein storage granules in the cytoplasm. **(C)** Upon resumption of growth after quiescence, Blm10 can act as nuclear transport receptor binding to 20S or 26S holo proteasomes. Blm10 import complexes are dissociated by RanGTP in the nucleus. The re-import of free 19S RP into the nucleus is not entirely understood.

No putative cNLS was identified within the RP lid subunits suggesting that the cNLS is conferred by a transiently bound protein during nuclear import. A candidate protein could be the cNLS-containing and Srp1-interacting Sts1 which reacts with the RP lid subunit Rpn11 in yeast two-hybrid (Figures 2, 4A). Both *RPN11* and *STS1* are suppressors of the *srp1-49* mutation (Tabb et al., 2000). Intriguingly, in *sts1*Δ*NLS* mutants the RP lid did not interact with Srp1. Cytoplasmic retention of the RP lid, base and the CP was observed, suggesting that Sts1 is required for the nuclear localization of proteasome holo-enzymes (Chen et al., 2011). Sts1/Cut8 is proposed to target the proteasome to the nuclear side of the NE (Tatebe and Yanagida, 2000; Takeda and Yanagida, 2005; Takeda et al., 2011). However, the extremely short half-life of Sts1 disputes a role as a scaffold protein for proteasome tethering to the NE. It rather serves as a transient adaptor protein and confers a cNLS to the RP lid during nuclear import. Sts1 is rapidly degraded, possibly when proteasome holo-enzymes are assembled. The association of Sts1 to Srp1 is also coupled with proteasomal degradation of nascent polypeptides in the nuclear periphery (Ha et al., 2016).

## Nuclear Import of Proteasome Holo-Enzymes

In dividing yeast cells the concept for nuclear import of proteasomes via the canonical pathway is based on modules, namely CP precursor complexes either as half-CP or pre-holo-CP, the RP base and lid which are assembled into holo-enzymes in the nucleus (Figure 4; Lehmann et al., 2002; Wendler et al., 2004; Isono et al., 2007). However, the assembly of proteasome holo-enzymes can also be completed in the cytoplasm before nuclear import, specifically upon exit from quiescence (Pack et al., 2014). Intriguingly, the CP does not seem to be transported on its own. We assume that the CP could degrade nuclear pore proteins on its passage through the NPC, since nuclear pore proteins belong to the family of intrinsically disordered proteins (Denning et al., 2003), favored substrates of the CP which are degraded in an ATP- and ubiquitin-independent manner (Tsvetkov et al., 2009).

To address the question how proteasome holo-enzymes are imported into the nucleus of living yeast cells, non-invasive fluorescence correlation spectroscopy (FCS) was conducted to investigate the spatio-temporal dynamics of proteasome holo-enzymes. First, the distribution of CP, RP base and lid was monitored in exponentially growing yeast cells by expressing GFP-labeled reporter subunits behind the endogenous promoter at the chromosomal locus (α4 for CP, Rpn1 for RP base, and Rpn7 for RP lid). A similar overall distribution was determined for all three subunits reflecting equal stoichiometry with 140–200 nM in the cytoplasm and 830–980 nM in the nucleus. Second, dual color fluorescence cross correlation spectroscopy (FCCS) revealed stable proteasome holo-enzymes that were labeled with differently colored fluorescent proteins fused to either CP and RP subunits. Compared with wild-type, their distribution was shifted from the nucleo- to the cytoplasm in *srp1-49* mutants, confirming that nuclear import of proteasomes follows the canonical pathway (Pack et al., 2014).

Finally, genetically tethered RP-CP-RP were analyzed by FCS. Since it is impossible to distinguish whether RP-CP-RP were assembled in the cytoplasm or nucleoplasm, their nuclear import was monitored in yeast cells upon exit from quiescence. For this purpose, yeast cells were grown to stationary phase. Proteasomes were depleted from the nucleus and accumulated in cytoplasmic storage granules. With the resumption of growth, the proteasome storage granules rapidly clear and genetically tethered RP-CP-RP were detected in the nucleus, providing evidence that cytoplasmic RP-CP-RP can pass the NPC into the nucleus (Pack et al., 2014).

Interestingly, yeast cells having proteasomes genetically anchored to the plasma membrane or to ribosomes resulted in conditional depletion of nuclear proteasomes and lethality, while yeast cells having proteasomes genetically anchored to the nucleus resulting in the depletion of cytoplasmic proteasomes show mild growth defects (Tsuchiya et al., 2013). Thus, cytoplasmic proteasomes are dispensable for cell growth but the major place for proteasomal proteolysis resides in the nucleus of proliferating cells. As mentioned in the introduction important classes of nuclear substrates are cell cycle regulators and transcription factors, e.g., Rpn4 which augments the transcription of proteasomal genes and orchestrates the expression of hundreds of genes involved in DNA repair and stress response (Jelinsky et al., 2000).

## Nuclear Import of Proteasomes upon Exit from Quiescence

With the transition from proliferation to quiescence proteasomes are exported from the nucleus into the cytoplasm, which seems to be independent of the canonical exportin Crm1/Xpo1 [(Nemec et al., 2017), our unpublished results]. In prolonged quiescence, ~90% of the proteasomes are depleted from the nucleus and sequestered into proteasome storage granules (PSG), which migrate as stable entities through the cytoplasm in quiescent yeast [Figure 4B; (Laporte et al., 2008)]. With the resumption of growth, PSG-resident proteasomes are imported into the nucleus within a time frame of few minutes in which proteasomes cannot be assembled from newly synthesized subunits (Figure 4C). Here, it is important to note that in quiescent cells proteasome holo-enzymes dissociate due to the decreased ATP level (Bajorek et al., 2003) and the lack of substrates. Upon the availability of glucose with the resumption of growth, the ATP level increases and short-lived proteins regulating cell cycle progression arise as proteasomal substrates which engage the assembly of proteasome holo-enzymes for protein degradation.

Notably, Blm10, a conserved 240 kDa protein of the HEAT repeat-like family, is bound to the CP and required to sequester the CP into the PSG. With the resumption of cell growth, Blm10 facilitated nuclear import of matured CP (Weberruss et al., 2013), thus seems to fulfill a dual function as importin and as chaperone of PSG formation.

Several pieces of evidence supported our conclusion that Blm10 represents the first CP-dedicated nuclear transporter (Figure 4C; Weberruss et al., 2013). Blm10 binds nuclear pore proteins and RanGTPase Gsp1. As described above for the concept of the canonical nuclear import the cargo dissociates from the β-importin, once it encounters RanGTP. Since Blm10-bound CP is dissociated by RanGTP (Weberruss et al., 2013), Blm10 behaves like Kap95, the canonical importin β. Moreover, Blm10 and Kap95 show structural similarities within members of the β importin family (Huber and Groll, 2012). Blm10 does not only recognize mature CP as import cargo but also incompletely matured pre-holo-CP and CP precursor complexes (Lehmann et al., 2008). Comparable with importin αβ, Blm10 recognizes import-competent CP cargoes by open α rings, which are either constitutively open or disordered (Fehlker et al., 2003; Li et al., 2007; Lehmann et al., 2008). Thus, Blm10 may have a backup function in nuclear import of CP precursor complexes as an alternative adaptor to the cNLS dependent pathway, especially when α ring gating is disturbed by genetic manipulations or stress. Possibly, Blm10 prevents the degradation of intrinsically disordered nuclear pore proteins by CPs with disturbed α ring gating when they pass through the NPC.The latter might explain why Blm10 remains associated with the CP in quiescence (Schmidt et al., 2005). Without Blm10 quiescent cells are hypersensitive toward DNA damage and proteotoxic stress and compromised in their fitness during aging (Doherty et al., 2012).

Quiescent *blm10*Δ mutants also fail to quickly resume cell growth which correlated with delayed nuclear import of the CP. Due to the lack of Blm10 in *blm10*Δ mutants CP precursor complexes must first be assembled as import cargoes to be transported by the canonical cNLS pathway.

It is still unknown how the RP is transported from the PSG into the nucleus upon exit from quiescence. Possibly nuclear import of the RP is facilitated by Spg5 which assists in the sequestration of the RP into PSG (Hanna et al., 2012; Marshall and Vierstra, 2018). Rpn2 of the RP base has an α-solenoid fold comparable with importin β and belongs to the family of HEAT-repeat proteins (Kajava et al., 2004; Huber and Groll, 2012). Thus, Rpn2 could serve as nuclear import receptor of the RP or mediate an interaction with importin β.

## Unexplored Topics in Proteasome Transport and PSG Formation

Post-translational modifications, such as phosphorylation, regulate the activity of nuclear proteasomes (Bose et al., 2004; Sha et al., 2011). Phosphorylation of proteasomal subunits enhances proteasomal proteolysis of misfolded proteins and modifies the cellular susceptibility to proteotoxic stress and protein aggregation (Collins and Goldberg, 2017; Marquez-Lona et al., 2017). We observed that PSG formation is disturbed in *snf1*Δ null mutants. Snf1 is an AMP-dependent kinase which senses glucose availability and seems to be involved in targeting the CP and RP into PSGs (Weberruss et al., 2013). *N*-myristoylation and N-acetylation of proteasomal subunits contribute to nuclear proteasome localization and suggest that multiple factors influence nuclear transport of proteasomes (Kimura et al., 2012; van Deventer et al., 2015).

As mentioned above nuclear export of proteasomes is still an unexplored topic.

## Author Contributions

CE wrote the manuscript, PW added critical comments on the manuscript, prepared the Figures and Figure Legends.

### Conflict of Interest Statement

The authors declare that the research was conducted in the absence of any commercial or financial relationships that could be construed as a potential conflict of interest.
